# A Case Report of Concurrent Epidermal Growth Factor Receptor (EGFR) Exon 18 (G719A) and Exon 21 (L833_V834delinsFL) Mutations and Treatment Challenges

**DOI:** 10.7759/cureus.70896

**Published:** 2024-10-05

**Authors:** Muhammad Hussain, Nicholas Mackrides, Stacey Su, Anjali Seth

**Affiliations:** 1 Pathology and Laboratory Medicine, Temple University Hospital, Philadelphia, USA; 2 Hematopathology, Fox Chase Cancer Center, Philadelphia, USA; 3 Thoracic Surgery, Fox Chase Cancer Center, Philadelphia, USA

**Keywords:** compound mutations, egfr mutations, next generation sequencing (ngs), pulmonary adenocarcinoma, tyrosine kinase receptor inhibitors

## Abstract

Molecular profiling of lung tumors is crucial for guiding targeted therapeutic strategies and identifying potential resistance mechanisms to specific therapies, such as epidermal growth factor receptor (EGFR) tyrosine kinase inhibitors (TKIs). During this profiling, mutations with uncertain treatment implications can be identified. This case study represents a 69-year-old female with a co-occurring EGFR mutation profile that presents a unique therapeutic challenge. Tumor DNA was used for next-generation sequencing (NGS) of a custom 275 cancer-related QIAseq Human Comprehensive Cancer Panel (Qiagen). Next-generation RNA sequencing was performed using the Illumina TruSight panel. FISH analysis and PD-L1 22C3 immunohistochemical testing were also performed. Microscopic analysis revealed an invasive adenocarcinoma with papillary, acinar, and focal micropapillary features with a 6 mm invasive component. The final pathology stage was determined to be pT1aN0M0. NGS for DNA variant detection identified two mutations in EGFR, an EGFR G719A and EGFR L833_V834delinsFL with a variant allele frequency (VAF) of 22.2% and 21.1%, respectively. Targeted NGS RNA fusion analysis was also performed, which came back negative. PD-L1 22C3 immunohistochemical testing showed only 1% of the tumor cells expression. FISH analysis revealed one copy of MET and D7Z1 in 27% of cells, indicating an aneuploid neoplastic clone with monosomy 7. EGFR TKIs are universally accepted as a first-line treatment for advanced non-small cell lung cancer (NSCLC) patients with a sensitizing EGFR mutation. While mutations such as G719A are sensitive to all generations of EGFR-TKI, the effects are unknown for rare compound mutations in EGFR, such as EGFR L833_V834delinsFL. There are no reports in the literature with any mention of an algorithm of treatment for such a case. The patient had two metachronous lung primary cancers resected in 2022 and 2024. Due to the complete surgical resection, the sensitivity of this mutation of TKIs could not be established. This unique mutation profile still remains of paramount importance to understand if the patient relapses or presents with a new tumor with the same genetic profile.

## Introduction

Epidermal growth factor receptor (EGFR) gene mutations are prevalent driver mutations in non-small cell lung cancer (NSCLC), particularly adenocarcinomas [1]. Molecular profiling of lung tumors is crucial for guiding targeted therapeutic strategies and identifying potential resistance mechanisms to specific therapies, such as EGFR tyrosine kinase inhibitors (TKIs) [2-4]. During this profiling, mutations with uncertain treatment implications can be identified. This case study represents a 69-year-old female with a co-occurring EGFR mutation profile that presents a unique therapeutic challenge.

The patient harbors a mutation in exon 18 (c.2156G>C, p.G719A), which is a potentially targetable alteration with the FDA-approved TKI Afatinib [3]. However, this case becomes particularly interesting due to the concurrent presence of a rare mutation in exon 21 (c.2499_2500delinsTT, p.L833_V834delinsFL). While previously reported in small-cell lung carcinoma (SCLC), the clinical significance of this specific mutation co-occurring with an EGFR exon 18 mutation in adenocarcinoma remains unknown. The efficacy of Afatinib or other TKIs in this specific context (G719A mutation with L833_V834delinsFL co-mutation) is unclear, highlighting the need for further investigation.

## Case presentation

**Figure 1:**
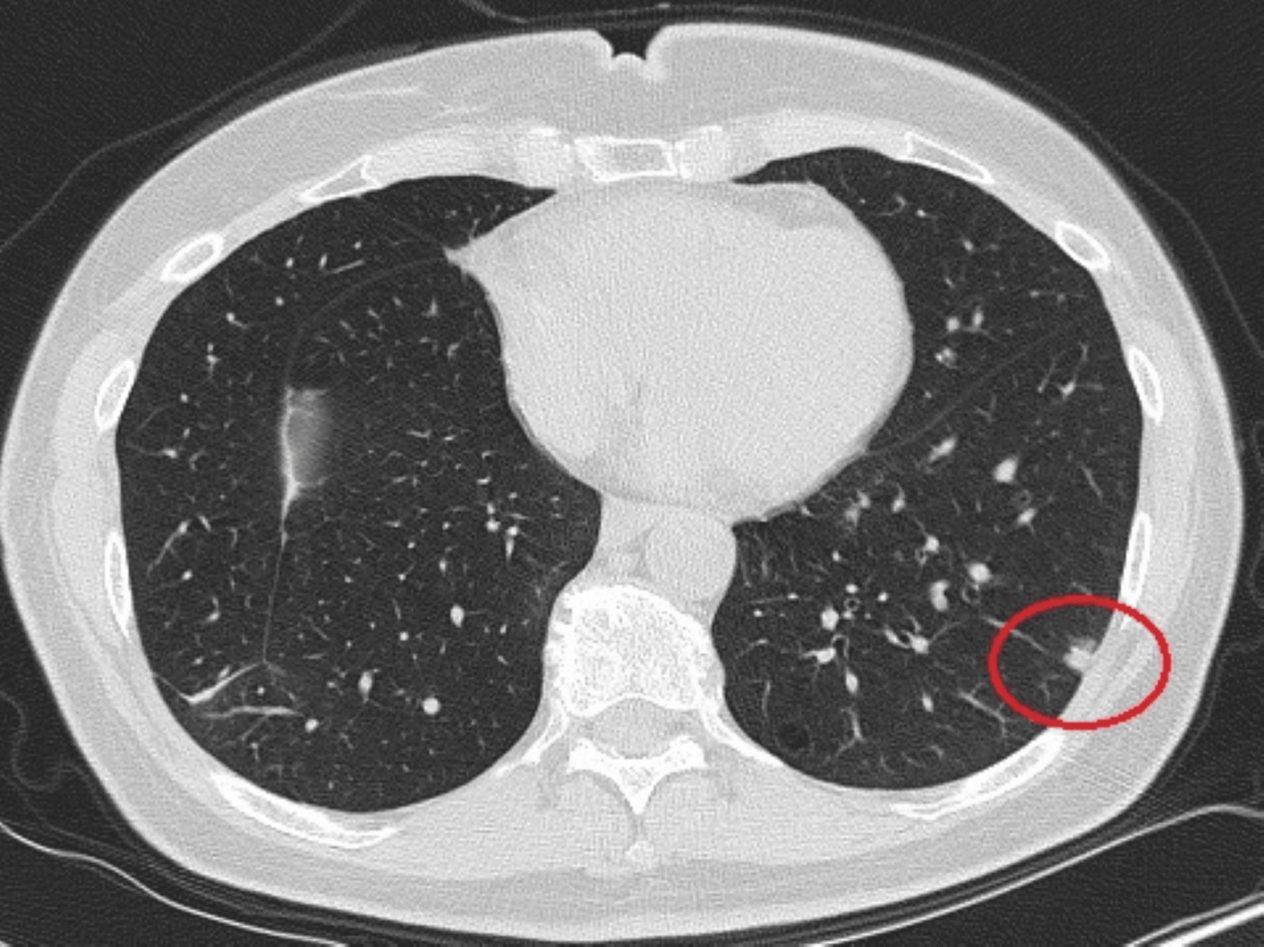
Subpleural left lower lobe 8 mm nodule with mild peripheral ground-glass density, dominant 5x5 mm solid component.

The patient is a 69-year-old woman with a smoking history of 30 pack-years who underwent right upper lobectomy in January 2022 for complete resection of a 2.3 cm minimally invasive adenocarcinoma (positive for TTF1 and Napsin A) amid a background of bilateral ground-glass lung nodules. During surveillance imaging, she was noted to have slow enlargement of a subcentimeter part-solid nodule in the left lower lobe concerning second primary lung cancer. A CT scan revealed an 8 mm subpleural nodule with a dominant 5x5 mm solid component and mild peripheral ground-glass density in the left lower lobe (Figure 1). Additionally, scattered sub-centimeter pure ground-glass nodules in the lungs were present, unchanged since at least December 2022.

**Figure 2:**
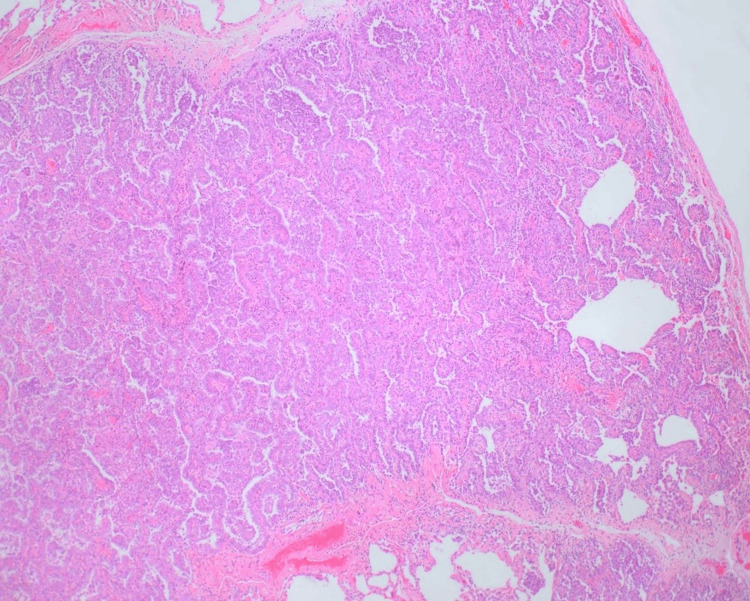
Low-power image illustrating the architectural pattern of papillary-type invasive adenocarcinoma, with acinar, lepidic, and focal micropapillary components.

**Figure 3:**
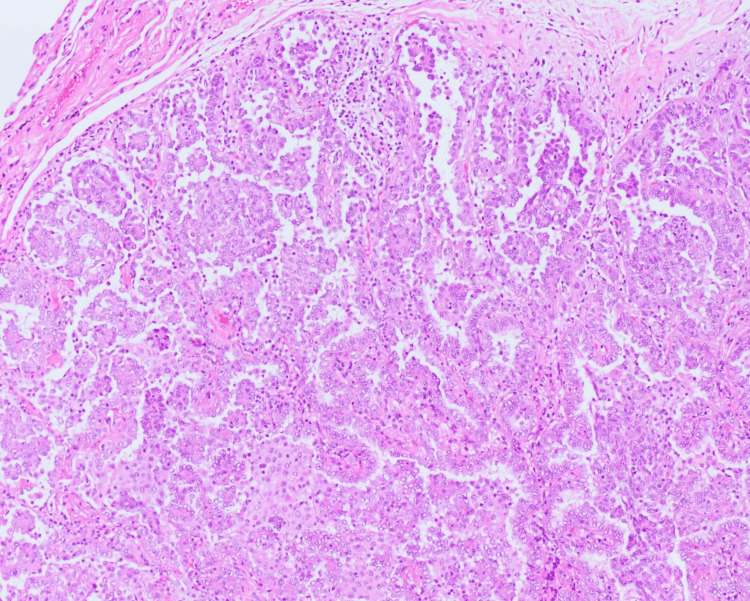
High-power image illustrating the architectural pattern of papillary-type invasive adenocarcinoma, with acinar, lepidic, and focal micropapillary components.

Video-assisted thoracoscopic wedge resection of the nodule was performed. Gross examination identified an 8 mm gray-tan subpleural nodule that was 1.4 cm away from the nearest resection margin. Microscopic analysis revealed an invasive adenocarcinoma with papillary, acinar, and focal micropapillary features with a 6 mm invasive component (Figures 2, 3). The final pathology stage was determined to be pT1aN0M0.

In her most recent follow-up visit, three months post-surgery, she reported doing well, although she still has residual chest pain, which has improved significantly from before. She also reported chronic stable dyspnea on exertion. Pulmonary function tests show mild obstructive physiology, mildly impaired diffusion, and small airway obstruction.

**Figure 4:**
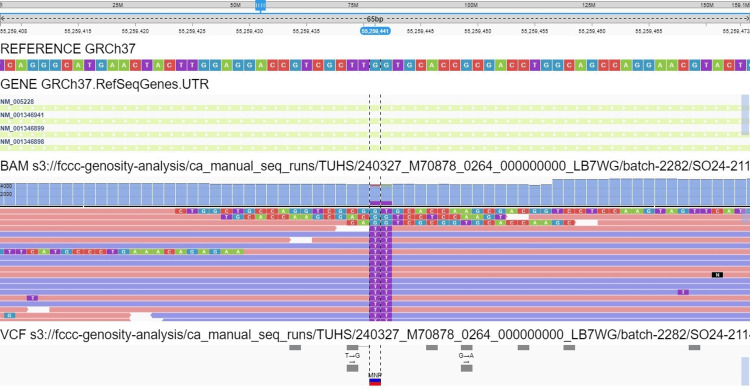
Visualization of the EGFR mutation EGFR L833_V834delinsFL by NGS. NGS, next-generation sequencing; EGFR, epidermal growth factor receptor

Given the metachronous lung primary adenocarcinoma amid a background of multifocal ground glass nodules suggesting the possibility of multifocal disease, a molecular profile was obtained to refine treatment options and predict targeted therapy response. Tumor DNA was microdissected and mutational analysis of the most common genes implicated in adenocarcinoma was performed by single-gene Sanger sequencing. No mutations in BRAF and KRAS were identified. However, two mutations were identified in EGFR, namely an exon 18 mutation (c.2156G>C, p.G719A) and a compound mutation in exon 21 (c.2499_2500delinsTT, p.L833_V834delinsFL) (Figure 4).

To explore and confirm this mutation further, next-generation sequencing (NGS) for DNA variant detection was performed. Paired normal and tumor DNA were used for multiplex amplification with gene-specific primers targeting the coding exons and exon flanking regions of 275 cancer-related genes using the QIAseq Human Comprehensive Cancer Panel (Qiagen). NGS was performed using the NextSeq2000 (Illumina) and analyzed with CLC Genomics Workbench (Qiagen). The variants were filtered based on variant allele frequency (VAF, ≥5%), mutation databases, and population-based reference datasets including gnomAD, ClinVar, and COSMIC. NGS confirmed this mutational change with EGFR G719A having a VAF of 22.2% and EGFR L833_V834delinsFL having 21.1%. The EGFR G719A mutation is known to be oncogenic and has an FDA-approved targeted therapy option. However, the significance of the EGFR L833_V834delinsFL mutation remains unclear.

**Figure 5:**
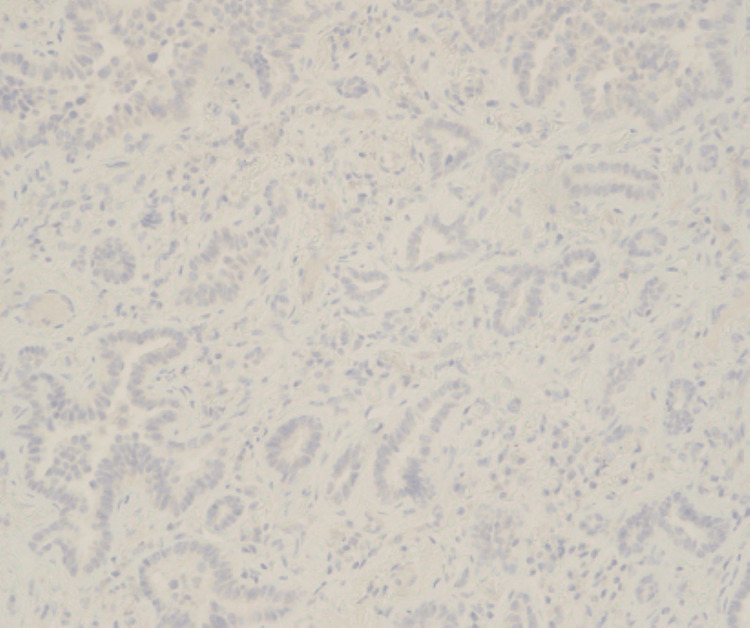
PD-L1 (clone 22C3) immunohistochemical staining reveals faint staining in 1% of tumor cells (400x magnification).

Targeted NGS RNA fusion analysis was also performed using a custom Illumina TruSight 523 gene panel, which resulted as negative. To narrow down the targeted therapy further, PD-L1 22C3 immunohistochemical testing was also performed, which showed only 1% of the tumor cell expression (Figure 5). FISH analysis revealed one copy of MET and D7Z1 in 27% of cells, indicating an aneuploid neoplastic clone with monosomy 7. Following these findings, treatment options were discussed.

## Discussion

Activating mutations in receptor tyrosine kinases play an important role in oncogenesis [5-8]. EGFR mutations have been reported in 11-52% of NSCLC cases and are found more commonly in East Asian patients compared with other ethnicities [9-13]. We describe a case with two co-occurring EGFR mutations, G719A and a rare L833_V834delinsFL mutation (Figure 3).

EGFR G719A is a missense mutation within the protein kinase domain of the EGFR protein (UniProt). This exon 18 mutation has been reported to result in ligand-independent activation of the EGFR protein and increased cell growth compared with wild-type EGFR [12,14]. The presence of a sensitizing EGFR mutation in a tumor is the strongest biological predictor of sensitivity to an EGFR TKI. Compared with conventional chemotherapy, EGFR TKIs have been shown to improve progression-free survival in NSCLC patients whose tumors harbor EGFR mutations [5,15-18]. The EGFR TKI Afatinib is FDA-approved for the treatment of patients with EGFR G719 mutant NSCLC.

EGFR L833_V834delinsFL appears in exon 21 and results in the deletion of two amino acids and the insertion of one amino acid in the kinase domain of the EGFR protein. This alteration has been reported (COSMIC, Dec 2021), but it has not been functionally characterized (PubMed, Dec 2021) and its effect on protein function is unknown [5,7,9,11,19]. However, it is known that individual mutations at these loci are oncogenic or likely oncogenic.

The EGFR exon 21 L833V mutation occurs in the EGFR tyrosine kinase domain. This mutation has been found in NSCLC [7-9]. Across three studies, patients with NSCLC harboring the EGFR L833V mutation in combination with the TKI-sensitizing EGFR L858R, G719A, or H835L mutations had a partial response (two of five), complete response (one of five), or stable disease upon treatment with gefitinib [7-9]. Furthermore, in an analysis of 860 patients with lung adenocarcinoma who were prospectively tested for actionable mutations, one patient (100%) with an EGFR L833V mutation in conjunction with an EGFR L858R substitution mutation who received EGFR TKI therapy and chemotherapy had clinical benefit [10]. 

The EGFR exon 21 L833F mutation is also located in the EGFR tyrosine kinase domain. This mutation has yet to be functionally characterized. Patients with lung cancer harboring the L833F mutation have had mixed responses to targeted therapies. Preclinical models with the L833F mutation show high sensitivity to first, second, third-generation, and Ex20 insertion-active EGFR inhibitors [11]. One patient had a partial response to chemotherapy but had progressive disease to an EGFR TKI [12]. Another patient had progressive disease despite being treated with erlotinib [13]. Another patient with G719A and L833F mutations had severe adverse effects in response to gefitinib [8]. One patient with the L833F mutation and an L858R mutation had clinical benefit in response to erlotinib [10]. 

The EGFR V834I mutation lies in the kinase domain of the protein. Expression of this mutation in the NIH3T3 cell line demonstrated it is likely activating as measured by slightly increased focus formation compared to wild-type EGFR [13]. 

The efficacy of EGFR-TKI in patients with NSCLC is related to EGFR mutations and is not well understood in patients with more than one mutation [6]. While the EGFR TKIs erlotinib, afatinib, gefitinib, dacomitinib, and osimertinib are FDA-approved for the treatment of patients with non-small cell lung harboring select EGFR-mutations such as G719A, their clinical utility in patients with EGFR L833_V834delinsFL altered NSCLC is unknown.

Our patient underwent resection of two separate stage IA lung adenocarcinomas. The standard of care for the treatment of stage IA NSCLC is resection alone. The clinical trials of targeted therapies only studied patients with stage IB-III NSCLC. Thus, none of the standard-of-care treatment guidelines recommend the use of targeted agents or other systemic treatment in patients with stage IA NSCLC.

This patient recently underwent a six-month surveillance CT chest scan, which shows no evidence of disease. She will continue to undergo six-month interval scans for at least three years before the surveillance scans will be extended to yearly.

## Conclusions

EGFR TKIs are universally accepted as a first-line treatment for advanced NSCLC patients with a sensitizing EGFR mutation. While mutations such as G719A are sensitive to all generations of EGFR-TKI, the effects are unknown for rare compound mutations in EGFR, such as EGFR L833_V834delinsFL. To the best of our knowledge, we believe this is the first case reported in the literature harboring these two mutations. Therefore, there are no reports in the literature with any mention of an algorithm of treatment for such a case. The patient had two metachronous lung primary cancers resected in 2022 and 2024. Due to the complete surgical resection, the sensitivity of this mutation of TKIs could not be established. This unique mutation profile still remains of paramount importance to understand if the patient relapses or presents with a new tumor with the same genetic profile.
